# Cross-sectional study of the anthropometric characteristics of children with congenital Zika syndrome up to 12 months of life

**DOI:** 10.1186/s12887-020-02365-6

**Published:** 2020-10-14

**Authors:** Rita de Cássia Oliveira de Carvalho-Sauer, Maria da Conceição Nascimento Costa, Enny S. Paixão, Natanael de Jesus Silva, Florisneide Rodrigues Barreto, Maria Gloria Teixeira

**Affiliations:** 1 Bahia State Health Secretariat, Epidemiological Surveillance Service of the East Regional Health Center, Avenida Esperança, 406, Santo Antônio de Jesus, Bahia ZC 44435-500 Brazil; 2 Institute of Collective Health, Federal University of Bahia, Rua Basílio da Gama, s / n. Canela, Salvador, Bahia ZC-40.110.040 Brazil; 3 London School of Hygiene and Tropical Medicine, London Keppel St, Bloomsbury, London, WC1E 7HT UK; 4 Centre for Data and Knowledge Integration for Health (CIDACS), Gonçalo Moniz Institute, Oswaldo Cruz Foundation (FIOCRUZ), Parque Tecnológico da Bahia. Rua Mundo, 121 – Trobogy, Salvador, Bahia ZC 41745-715 Brazil

**Keywords:** Zika virus infection, Microcephaly, Anthropometry

## Abstract

**Background:**

Little is known about physical development of children with Congenital Zika Syndrome (CZS). This study aims to evaluate the anthropometric characteristics of children with CZS up to 12 months.

**Methods:**

This is a cross-sectional study developed with 46 children with CZS living in Bahia. We used the Public Health Events Register, Live Births Information System and Childcare Records of Primary Health Care Services. Descriptive analysis was performed by distributing absolute and relative frequencies and median and interquartile range. The Weight/Age (W/A), Length/Age (L/A), Weight/Length (W/L) and Head Circumference/Age (HC/A) indexes were calculated for each month and expressed in z-score values, and the results were evaluated individually and by group average. Values between ≥ − 2 and ≤ 2 standard deviations were used as reference. T-Student and Spearman’s Correlation Tests were applied to verify the existence of any relationship between maternal and children’s variables with the anthropometric indexes weight/age and height/age at birth and at 3, 6 and 12 months of age.

**Results:**

The studied children had high proportions of low birth weight (23.9%), dysphagia (56.8%) and seizures (53.5%). The mean z-score for the HC/A index at birth was − 3.20 and remained below − 3 z-scores throughout the assessed period. The analysis of the indices equivalent to every single child’s anthropometric measurement showed a deficit in 20.4% of the W/A, 39.1% of the L/A, 9.2% of the W/L and 85.7% of the HC/A measurements. Distribution of the mean values of these anthropometric indices revealed a risk of delayed stature growth (L/A < -1 z-score). There was a statistically significant association between L/A at 12 months and dysphagia (*p* = 0.0148) and a positive correlation between breastfeeding time and W/A. No statistically significant correlation was found between any other tested variables.

**Conclusions:**

We observed a deficit in the HC/A index, which is a common feature in CZS, but also a high proportion of W/A and L/A deficit. The average group z-score highlighted the risk of delay in stature growth for age, which calls attention to the need for health interventions, as this condition exposes them to a higher risk of morbidity and mortality.

## Background

The potential causal association between Zika virus (ZIKV) infection during pregnancy and congenital malformations was raised in 2015, after an unusual increase in the number of births of children with microcephaly in Brazil, months after an unprecedented epidemic produced by this virus [1]. Next, a broad spectrum of congenital malformations and dysfunctions associated with prenatal ZIKV exposure suggested the detection of a new syndrome, called “Congenital Zika Syndrome” (CZS) [2].

The long-term effects on the growth and development of children with congenital ZIKV exposure are not yet well known. So far, some of the main reported clinical abnormalities are low birth weight, arthrogryposis, heart disease, microcephaly with a specific pattern of brain damage, delay in neuromotor and cognitive development, impaired vision and hearing, seizures, dysphagia and failure to thrive [3–7].

In general, a higher nutritional risk is observed among children with mental disabilities and/or neurological diseases [8–10]. In particular, children with CZS possess some elements that can constitute important barriers to adequate nutrition, especially through the combination of neurological and oral-maxillofacial disorders. Derangement such as cervical hypotonia and appendicular hypertonia, hyperexcitability with extremely irritated and hard-to-sooth crying, seizures that over time can become more resistant to drug treatment [11], potentially impair the maintenance of an adequate dietary routine for these children. Additionally, multiple defects of the oral and maxillofacial structure such as narrow palate form, abnormal insertion of the upper labial frenulum, posterior lingual frenulum, tongue anterior projection, delayed dental eruption and oral escape, harm the oral phase of food and are often associated with presence of dysphagia [12, 13]. This can be characterized by significant impairment of the oral phase of swallowing, prolonged time for the preparation of the bolus, delays at the beginning of the pharyngeal swallowing phase and in the emptying of the distal esophagus, besides evidence of a higher risk of aspiration and penetration of food into the airways, typical of severe dysphagia [14].

Considering that most children with CZS in Brazil come from poorer families [15], socioeconomic difficulties can exacerbate the health risks, in addition to those already determined by CZS itself.

The critical relationship between food and nutrition with children’s growth and physical development is widely recognized [16, 17], however, to date, there have been few studies characterizing the growth of children with CZS [18], which is why it is still necessary to increase the evidence on the real impact of this syndrome on indicators of physical development. This study aims to assess the anthropometric characteristics of children with CZS at birth and up to 12 months of life.

## Methods

A cross-sectional descriptive study was carried out in 22 municipalities in the State of Bahia, Brazil. Data collection took place from May to November 2017. Children were selected by convenience sampling, as they were residents in municipalities which had operational feasibility for data collection by researchers, and authorization from Health managers or directors to access health information. The inclusion criteria in this study were: a) to be resident in one of the participating municipalities, be notified in the RESP and classified as a confirmed or presumed case of CZS, through a complete clinical and epidemiological investigation; b) to have anthropometric assessments of weight, length or head circumference, until 12 months of age, in the medical records of the primary care service of their place of residence. Children classified as confirmed cases of CZS, but who had no anthropometric record in the first year of life found in the medical records of the primary care service were excluded from the present study.

RESP – Registro de Eventos em Saúde Pública (Public Health Event Registration) is an electronic form from the Ministry of Health of Brazil (MoH), where notifications of suspected cases of CZS and respective clinical and epidemiological investigations are recorded, and the classification of each notification occurs when sufficient elements are gathered to classify these suspected cases based on specific guidelines [19]. Notification of suspected CZS cases is mandatory for all public and private health services in Brazil. Children whose mother had a history of suspected or confirmed Zika infection during pregnancy, or who at birth had a cranial circumference less than − 2 standard deviations for gestational age at birth and sex (according to the InterGrowth table [20]), or craniofacial disproportion, or arthrogryposis, or neurological, visual or auditory manifestations, or alteration of neuropsychomotor growth and development not explained by other causes can be considered suspected cases of CZS. The suspicion of CZS is confirmed by laboratory criteria when there is a positive or reagent result for the Zika virus in RT-PCR or serology test, in a sample of the child, provided that the quality requirements for the test, and result are negative or inconclusive in at least 1 STORCH (syphilis, toxoplasmosis, rubella, cytomegalovirus or herpes simplex) in a sample of the child or the mother (during pregnancy). Children who meet the epidemiological, clinical and image examination criteria for suspected CZS, with negative laboratory results for STORCH and who for some reason were unable to undergo the laboratory diagnosis of ZIKV, are considered presumed cases of CZS.

In addition to the RESP, data sources for this study were also SINASC – Sistema de Informação sobre Nascidos Vivos (Live Births Information System) and Childcare Records of Primary Health Care Services. From the SINASC (state database) we obtained maternal sociodemographic and gestational data, and date of birth, sex, weight, and length of child, at birth. From childcare records, in primary care services, we obtained children’s clinical data, including all available records of anthropometric assessments.

In primary care services in Brazil, children’s anthropometry is usually performed by nurses or nursing technicians, following the techniques recommended by the MoH for children under 2 years old [21]. They are as follows: a) for weight measurement, the child should be completely naked and placed on a pre-calibrated balance, on a flat, firm surface. b) regarding body length, the child should be placed supine, on a smooth, firm and horizontal surface, with arms parallel to the body, and head, back, buttocks and heels firmly supported on the surface, with the knees extended. Then, the distance between the top of the head and the sole of the feet is measured with an infantometer. c) to measure the head circumference, an inelastic tape measure is passed over the most prominent point at the back of the skull (occipital) and on the eyebrows, and the perimeter is read. The scales and infantometers used in these different primary care services varied in terms of brand and model. The maternal information collected were: race/color (white, black or mixed); age group (≤ 19 years old/teenager or ≥ 20 years old/adult); if she lives with the child’s father (yes/no); educational level (up to incomplete elementary school/complete elementary school until incomplete high school/complete high school or more); type of delivery (vaginal or cesarean); paid work (yes/no); type of pregnancy (single or twin); duration of pregnancy (< 37 weeks = preterm / ≥37 weeks = term or post-term); and number of children alive (≤2 or ≥ 3). The information collected regarding the child was: date of birth, sex (male/female), dysphagia (yes/no), seizures (yes/no), duration of breastfeeding (in months) and anthropometric data – weight (g), length (cm) and head circumference (cm), with respective measurement dates. The date of birth of the child and date of measurements of weight, length and head circumference were used to obtain anthropometric indices, according to age. For preterm children, the Intergrowth-21st software was used to calculate the weight/age (W/A), length/age (L/A) and head circumference/age (HC/A) indexes from birth to the age equivalent to 64 weeks after the date of the last maternal menstruation. After 64 weeks of the last maternal cycle, these indexes were calculated using WHO-Anthro software, which was also used to calculate the weight/length (W/L) index of preterm children and all anthropometric indices of term children, from birth to 12 months of age. The indices were obtained in z-scores, and the interval between z-scores ≥ − 2 and ≤ 2 standard deviation (SD) was considered as a reference, as recommended by the World Health Organization (WHO) and adopted by the MoH [21, 22].

Descriptive analysis was performed of the absolute and relative distribution of categorical variables, as well as the number of children with available anthropometric measures, for each month of life, and the proportion of those who had z-score values <− 2 standard deviations for each anthropometric index evaluated. The median and interquartile range of the variable “duration of breastfeeding” was calculated. Mean z-scores from the available measures, for each month of life, were calculated to verify the distribution of the W/A, L/A, W/L and HC/A indexes for the whole group of children evaluated. It is important to highlight that the number of children with available anthropometric measures varied with each month of life observed. The T-Student Test was applied to verify the association between the variables child’s sex, low birth weight, dysphagia or seizures with the W/A and L/A indexes at birth, at 3, 6 and 12 months of life. It was also verified if there was any correlation between head circumference at birth, duration of breastfeeding, as well as maternal variables age, race / color, education or duration of pregnancy with the child’s W/A and L/A indices at birth, at 3, 6 and 12 months of the life, through the calculation of Spearman’s correlation coefficient.

The project of this study was approved by the Research Ethics Committee of the Institute of Collective Health of the Federal University of Bahia and met the recommendations of Resolution 466/2012 of the National Council of Ethics in Research / CONEP for research in human (CAAE registration number no 2.102. 890).

## Results

At the beginning of data collection, there were 70 children (born between October 2015 and May 2017) classified as confirmed or presumed cases of CZS in the participating municipalities. Of these, 10 (14.3%) did not have records of medical care at the primary care service and another 14 (20.0%) children had records of child appointments, however, no anthropometric data was recorded up to 12 months of life. The remaining 46 (65.7%) cases comprised the study sample, with 44 presumed CZS cases and 2 confirmed CZS cases by positive laboratory examination for ZIKV.

Of the total sample, 54.3% were male, 23.9% were born weighing less than 2500 g, 13.0% were preterm, 2.2% twin, 15.2% were children of adolescent mothers, 56.8% had dysphagia, and 53.5% had seizures (Table 1). About 75.0% of the mothers lived with the child’s father, 75.7% declared themselves to be mixed race, 54.3% had completed high school or more, 31.8% had three or more children and 11.1% had paid work. The median duration of breastfeeding for the group of children, in their first 12 months of life, was 6.7 months (interquartile range = 4.5; 9.5), with a minimum of 0 months (not breastfed) and a maximum of 12 months (still breastfeeding at 12 months) – data not presented in the table. The mean z-score for the HC/A index at birth was − 3.20, and when stratified by sex, it was − 3.45 for boys and − 2.81 for girls.

**Table 1:** Number and percentage^a^ of children with Congenital Zika Syndrome, born between October 2015 and May 2017, according to sociodemographic and health characteristics. Bahia^b^, Brazil, November 2017

Characteristics		n	%
**Child**	**Sex**	**46**	-
	Female	21	45.7
	Male	25	54.3
	**Low Birthweight**	**46**	-
	No	35	76.1
	Yes	11	23.9
	**Dysphagia**	**44**	-
	No	19	43.2
	Yes	25	56.8
	**Seizures**	**43**	-
	No	20	46.5
	Yes	23	53.5
**Maternal**	**Race/Skin color**	**37**	-
	White	2	5.4
	Black	7	18.9
	Mixed	28	75.7
	**Age group (years)**	**46**	-
	Teenager (≤19)	7	15.2
	Adult (≥20)	39	84.8
	**Schooling**	**46**	-
	Up to incomplete elementary school	9	19.6
	Complete elementary school until incomplete high school	12	26.1
	Complete high school or more	25	54.3
	**Type of delivery**	**46**	-
	Vaginal	26	56.5
	Cesarean	20	43.5
	**Type of pregnancy**	**46**	-
	Single	45	97.8
	Twinning	1	2.2
	**Duration of pregnancy (weeks)**	**46**	-
	Term (≥37)	40	87.0
	Pre-term (< 37)	6	13.0

^a^It refers only to children with information available on each variable

^b^Only 22 municipalities

None of the children had a complete monthly record of anthropometric data, which made it impossible to assess the evolution of anthropometric indices over the first year of life, thus, a cross-sectional approach to the available data was carried out. A total of 226 weight-, 151 length- and 168 head circumference measurements were found in the medical records, which is equivalent to an average, per child, of 4.9 weight measurements (minimum 2; maximum 10), 3.3 length measurements (minimum 0; maximum 9) and 3.7 head circumference measurements (minimum 0; maximum 9), between birth and 12 months of life. Of the 226 available weight measurements, 46 (20.4%) corresponded to a critical z-score value less than − 2 standard deviations, for the weight/age index. Z-scores were also below − 2 standard deviations in 39.1% of the length/age single values, 9.2% of the weight/length single values and 85.7% of the head circumference/age single values, of this group of children (Table 2).

**Table 2:** Number (N) of children with Congenital Zika Syndrome^a^, with registered anthropometric measurements, and number (n) and proportion (%) of those who had deficit (z-score < − 2SD) for the Weight/Age (W/A), Length/Age (L/A), Weight/Length (W/L) and Head Circumference/ Age (HC/A), according to sex and age. Bahia^b^, Brazil, November 2017

Age (months)	Anthropometric indexes											
	W/A			L/A			W/L			HC/A		
	N	< -2SD		N	< −2SD		N	< −2SD		N	< −2SD	
		n	%		n	%		n	%		n	%
0^c^	45	9	20.0	28	11	39.3	22	5	22.7	34	26	76.5
1	25	2	8.0	16	5	31.3	15	1	6.7	20	17	85.0
2	13	3	23.1	8	5	62.5	8	1	12.5	9	9	100.0
3	15	3	20.0	10	5	50.0	10	1	10.0	12	10	83.3
4	17	5	29.4	9	3	33.3	9	-	-	14	12	85.7
5	17	5	29.4	13	4	30.8	11	1	9.1	12	9	75.0
6	19	4	21.1	11	4	36.4	11	-	-	13	13	100.0
7	12	2	16.7	8	3	37.5	8	-	-	6	4	66.7
8	10	4	40.0	6	4	66.7	6	1	16.7	7	6	85.7
9	18	4	22.2	13	4	30.8	13	1	7.7	15	13	86.7
10	12	3	25.0	8	4	50.0	8	-	-	8	8	100.0
11	8	-	-	7	3	42.9	7	-	-	6	6	100.0
12	15	2	13.3	14	4	28.6	14	2	14.3	12	11	91.7
**Total**	**226**	**46**	**20.4**	**151**	**59**	**39.1**	**142**	**13**	**9.2**	**168**	**144**	**85.7**

*SD* standard deviation, *N* number of children with available anthropometric measurements, at each age (in months), in medical records, *n* number of children with anthropometric index equivalent to a value less than − 2 SD according to age and sex

^a^Children born between October 2015 and May 2017

^b^Only 22 municipalities

^c^At birth

The mean values of the W/A, L/A, W/L indices of the children, at birth, were respectively, − 0.72, − 1.05 and − 0.43 z-scores. From the first month, until 12 months of life, the W/A mean values ranged between 0.16 and − 1.59 z-scores, while the L/A mean values ranged between − 1.11 and − 3.17 z-scores, and the W/L mean values ranged between 2.43 and − 0.43 z-scores. When stratified by sex, in general, the average values of z-scores for boys were lower than for girls, for the W/A, L/A and HC/A indices throughout the period evaluated (Table 3).

**Table 3:** Average of the z-score values for the indexes weight/age (W/A), length/age (L/A), weight/length (W/L) and head circumference/age (HC/A), according to age and sex of children with Congenital Zika Syndrome^a^. Bahia^b^, Brazil, November 2017

Age (months)	Anthropometric indexes											
	W/A			L/A			W/L			HC/A		
	Total	Male	Female	Total	Male	Female	Total	Male	Female	Total	Male	Female
0^c^	−0.72	−0.86	−0.56	−1.05	−1.36	− 0.63	− 0.43	0.13	−1.23	− 3.20	− 3.45	−2.81
1	− 0.83	− 1.02	− 0.59	− 1.27	− 1.60	− 0.83	0.49	0.94	− 0.02	−4.00	− 4.50	− 3.53
2	− 1.59	−2.02	− 0.91	−2.08	−2.71	− 1.02	0.36	0.40	0.30	−5.80	−6.63	−2.75
3	−0.45	− 0.57	− 0.31	− 1.47	− 1.97	−0.97	1.83	2.18	1.48	−4.10	− 4.52	−3.56
4	−0.93	− 1.07	− 0.83	− 1.23	− 2.22	− 0.74	1.24	1.73	0.99	− 5.10	− 6.61	−4.03
5	− 0.34	− 0.51	− 0.23	− 1.73	− 1.86	− 1.59	1.82	1.16	2.38	−4.00	− 4.55	−3.38
6	−0.27	− 0.93	0.86	− 1.81	−2.12	− 0.98	1.97	1.12	4.25	− 5.10	− 5.27	−4.58
7	−0.11	−1.23	0.46	−1.38	−2.22	− 1.10	2.43	1.43	2.76	−4.40	−5.50	−3.34
8	−1.39	−1.94	−0.85	− 3.17	− 3.27	−2.99	0.45	0.02	1.32	−6.50	−8.12	−4.27
9	−0.38	−0.43	− 0.34	−1.42	−1.46	− 1.38	1.23	0.91	1.61	−5.20	−6.02	−4.34
10	−0.50	−1.14	0.79	−1.93	−2.47	−1.01	1.18	0.74	1.91	−6.00	− 6.20	−4.79
11	0.16	0.30	−0.26	−1.11	− 1.42	− 0.33	1.20	1.75	− 0.17	−5.60	−6.04	−3.53
12	−0.14	− 0.49	0.57	−1.12	−1.16	− 1.05	1.00	0.60	1.71	−6.30	−6.17	−7.45

^a^Children born between October 2015 and May 2017

^b^Only 22 municipalities

^c^At birth

There was a statistically significant association between L/A at 12 months and dysphagia (*p = 0.0148*) – data not shown in the table, and a positive correlation between breastfeeding time and W/A at 3 (*r*_*s*_ = 0.7012; *p* = *0.0052*) and 6 (*r*_*s*_ = 0.5388; *p* = *0.0256*) months (Table 4). We also found a correlation between both age (*r*_*s*_ = 0.5293; *p* = 0.11565) and maternal race / color (*r*_*s*_ = 0.5477; *p* = 0.12687) with the L/A index of the child in the 3rd month of life, however, this had no statistical significance. All other tested variables showed a weak to negligible correlation with the W/A and L/A indexes at birth, in the 3rd, 6th and 12th months of life, without statistical significance (Table 4).

**Table 4:** Spearman correlation coefficient values between maternal and child’s variables and the anthropometric indices Weight/Age (W/A) and Length/Age (L/A) at birth, and at 3, 6 and 12 months of life of children with Congenital Zika Syndrome born between October 2015 and May 2017. Bahia^a^, Brazil, November 2017

	Weight/Age				Length/Age			
	At birth	At 3 months	At 6 months	At 12 months	At birth	At 3 months	At 6 months	At 12 months
***Breastfeeding time***	0.047	0.701*	0.539*	0.081	0.175	0.372	0.573	−0.057
***Head circumference at birth***	–	0.559	0.720	−0.415	–	0.481	0.624	0.182
***Mother’s age***	0.142	0.163	0.202	−0.404	− 0.006	0.529	0.493	0.298
***Mother’s color/race***	0.151	0.290	0.057	−0.048	0.080	0.548	−0.311	−0.500
***Mother’s schooling***	−0.043	0.377	0.168	−0.039	−0.126	− 0.080	0.236	0.485
***Duration of pregnancy***	−0.067	−0.343	− 0.309	−0.499	− 0.117	0.149	− 0.143	−0.088

^a^Only 22 municipalities

*p < 0,05

## Discussion

This study shows that a high proportion of the anthropometric assessments of this group of children indicate deficits, mainly those related to growth in height. The analysis of every single anthropometric assessment revealed high proportions of low weight and length for age, although the average values of the anthropometric indices are within the normal range for the z-score (≥ − 2 and ≤ 2 standard deviations). In addition, we observed that the average L/A values were always below − 1 z-scores, indicating a risk of delay in linear growth, and the analysis stratified by sex, showed that boys had lower mean values than girls, for the indices W/A, L/A and HC/A. The L/A index also showed an association with the presence of dysphagia. Similar findings were described by Oliveira et al. (2020), who found 33% low weight in children with CZS versus 4% in a control group [12], and Soares et al. (2019), who observed that the mean weight and length in the infants who were ZIKV exposed in pregnancy were significantly lower than in non-exposed children, in the control group, at 3 months of age [23]. Santos et al. (2019) [24] also highlighted a worsening of the nutritional status of children with microcephaly due to ZIKV after 12 months of life, compared to that presented at birth, and a significant correlation was identified between the values of HC/A z-scores and all anthropometric indices.

Another important observation in this group of children with a high proportion of W/A deficit was a positive correlation between breastfeeding time and the W/A index at 3 and 6 months of age. This was considered an expected result, probably related to the protective effect conferred by breastfeeding. On the other hand, this association was not observed at 12 months, but it is worth noting that the median duration of breastfeeding of these children was 6.7 months and the third quartile was 9.5 months, which means that a minority of these children still breastfed at 12 months, and this possibly explains the difference at this age. Agostoni et al. (1999) showed that breast fed groups had significantly higher growth indices at 1 month (W/A, L/A), 2 months (W/A) and 3 months (W/A, L/A) of age, compared to formula fed infants [25].

Information extracted from the Brazilian food and nutrition surveillance system showed that 134,913 children under 2 years old, residents of Bahia, were assessed anthropometrically in primary care in 2016, and the deficit ratio of anthropometric indices was 3.59% for W/A, 14.49% for W/L and 7.09% for W/L. In 2017, 133,676 children under 2 years were evaluated in Bahia, observing a 3.44% deficit in W/A, 15.91% in L/A and 6.61% in W/L [26]. Our data show how far away the children with CZS are from the expected anthropometric values, which respectively presented the proportions of 20.4, 39.1 and 9.2% for W/A, L/A, W/L < − 2.

Consistent with the literature [27], microcephaly was a common sign in this evaluated group. It is known that the reduction in head circumference in children with CZS is, in general, accompanied by neurological damage with impaired neuromotor functions [19], and a tendency to have seizures and dysphagia [4, 14, 28]. Although there were reports of these dysfunctions in more than a half of the children studied, the real magnitude of seizures and dysphagia in children with CZS may be even greater, given the possible underreporting of these diagnoses in the medical records of primary health care services. This is a worrying because dysphagia can lead to dehydration, malnutrition and bronchial aspiration pneumonia [29], and children who need to make chronic use of anticonvulsants may experience a reduction in waking hours, due to the drowsiness induced by medications, and this will hinder sufficient food consumption to supply their daily nutritional requirements.

Regarding the high proportion of low birth weight found in our study, it is highlighted that both intrauterine growth restriction and low birth weight have been reported in case series of children with congenital Zika syndrome [18]. This finding may be related to placental damage produced by prenatal ZIKV infection [30–32], which is another condition that can hamper the physical development of these children. It is worth mentioning that part of the studied group was born with a gestational age of less than 37 weeks, which may also have contributed to lower mean values of the z-score for anthropometric indices during the first year of life. This is because premature infants tend to maintain a lower weight and height throughout childhood compared to children born at term [33].

Understanding an individual’s nutritional status goes beyond elucidating digestive and metabolic processes, since access and consumption of food are also subject to socioeconomic, political, social representations, habits and beliefs, among other factors [34]. Almost half of the assessed mothers of children with CZS had less than high school education and only a small proportion had paid work. These factors, together with the increase in family expenses due to the itineraries and supplies for the treatment of the sick child, may interfere with the family’s financial capacity to preserve the child’s food and nutritional security. Thus, initiatives such as access to the Continuous Supply Benefit (Law n° 13.985, April 07 2020) [35], one of the Brazilian government’s social programs that grants a minimum monthly wage to the elderly and people with disabilities, and others such as multidisciplinary and comprehensive care in the health care network, with guaranteed nutritional support, in addition to specific health promotion and education actions to mitigate the problems arising from CZS, can positively contribute to more appropriate nutritional and health care, given the special needs of these children.

The limitations of this research include aspects such as the non-representativeness due to the selection of participants by convenience sample, unavailability of complete monthly registration of anthropometric data and a potential bias in the reporting of seizure, dysphagia and breastfeeding time in primary care records, as well as the possible lack of standardization of the measurement instruments (scales, measuring tape and infantometer) used in health services. All of these should be taken into consideration when interpreting the results of this study. Despite these, however, it is understood that the findings presented here are relevant to Public Health, as they corroborate the findings of other authors and contribute to strengthen the evidence of the possibility of anthropometric deficit during the first year of life of children with CZS, because these deficits substantially increase the nutritional and health risks [36]. This alert to the need for careful nutritional monitoring, as these problems can be minimized with appropriate interventions such as access to multidisciplinary health monitoring and the provision of nutritional support. They also highlight the urgency of the implementation the health care network for children with CZS, with a systematic record of anthropometric measures aiming at their regular growth and development monitoring. This will enable the carrying out of more robust longitudinal studies about the growth trajectory of the population affected by CZS and other conditions.

## Conclusions

This group of children with Congenital Zika Syndrome, up to 12 months old, presents a high proportion of weight deficit for age, and are at risk of delay in stature growth, which underlines the necessity for health interventions, given that this condition exposes them to a higher risk of morbidity and mortality.

## Data Availability

The data that support the findings of this study are available on request from the first author (email contact: rita.sauer@outlook.com) and ethical approval. The data are not publicly available due to restrictions as they contain information that could compromise the privacy of research participants.

## Abbreviations

CZS: Congenital Zika Syndrome
CAAE: Certificate of Presentation of Ethical Appreciation
CONEP: National Council of Ethics in Research
HC/A: Head Circumference/Age
L/A: Length/Age
MoH: Ministry of Health of Brazil
RESP: Public Health Events Register
SD: Standard Deviation
SINASC: Information System on Live Births
WHO: World Health Organization
W/A: Weight/Age
W/L: Weight/Length
ZIKV: Zika virus
